# Estimated levels of glycated albumin and C-reactive protein/total albumin ratio might distinguish prediabetics among the apparently healthy people and predict the vulnerability for development of steatohepatitis and cardiac risk

**DOI:** 10.37796/2211-8039.1639

**Published:** 2025-03-01

**Authors:** Rizk S. Sarhan, Raafat R. Mohammed

**Affiliations:** a Department of Internal Medicine, Faculty of Medicine, Benha University, Benha, Egypt; b Hospital Lab, Clinical Pathology Department, Faculty of Medicine, Benha University, Benha, Egypt

**Keywords:** Prediabetes, Glycated proteins, C-reactive protein, Cardiac risk, Hepatosteatosis, Insulin resistance

## Abstract

**Background:**

Prediabetes precedes Type-2 diabetes development, is characterized by impaired glucose tolerance (IGT) as judged by the 75-g oral glucose tolerance test (75-OGTT) and is associated with higher cardiac risk (CR), dyslipidemia, hepatosteatosis (HS) and cancer.

**Aims:**

The study aimed to determine the distinguishing ability of estimated levels of glycated albumin (GA) and hemoglobin A1c (HbA1c), and serum C-reactive protein (CRP) and serum total albumin (TA) levels for prediabetic out of apparently healthy subjects.

**Methods:**

IGT was diagnosed if fasting blood glucose (FBG) was 100–125 mg/dl and if < 100 mg/dl subject is normal glucose tolerant (NGT). CR, HS and insulin resistance (IR) were suggested if the calculated atherogenic index of plasma (AIP) was >0.1, HS index (HSI) was >36 with high computerized hepatorenal index (HRI) and the homeostasis model assessment of IR (HOMA-IR) score was >2, respectively.

**Results:**

253 subjects (50.8%) had IGT and were older, more obese and mainly females. IGT had higher GA%, HOMA-IR score, serum CRP and lower TA with high CRP/TA ratio (CAR) than NGT. The frequency of subjects had AIP>0.1, HSI>36 and high HRI was significantly higher among IGT subjects. Statistical analyses defined high GA% and CAR as predictors for IGT, HOMA-IR>36 and AIP score >0.1, while high CAR is the only predictor for HSI score >36 and high HIR score.

**Conclusion:**

Prediabetes is not uncommon and high GA% and CAR might differentiate them among the apparently healthy population, and could predict those at increased risk for IR, HS and at CR.

## 1. Introduction

Diabetes is a metabolic disorder that occurs secondary to defective carbohydrate metabolism due to either insulin resistance (IR) or insulin deficiency and is associated with dysfunction of pancreatic β-cell and is characterized by hyperglycemia [1].

Type-2 diabetes (T2D) is the most prevalent type and represents a worldwide serious public health problem. Insulin resistance and β-cell failure are the major components of T2D pathology with subsequent endoplasmic reticulum stress signaling due to glucolipotoxicity that in association with the β-cell dysfunction initiates the deleterious vicious cycle observed in T2D [2].

Prediabetes (PD) or intermediate hyperglycemia precedes T2D development and is characterized by impaired fasting blood glucose (IFBG) and glucose tolerance (IGT) as judged by the 75-g oral glucose tolerance test (OGTT) [3]. According to the American Diabetes Association (ADA), PD was defined as FBG ranging between 5.6 and 6.9 mmol/L and/or glycosylated hemoglobin A1c (HbA1c) level in a range of 5.7–6.4%, and according to the criteria of World Health Organization/International Expert Committee (WHO/IEC) as FBG of about 6.1–6.9 mmol/L and/or HbA1c of 6–6.4% [4]. However, another study concluded that both ADA and WHO definition of PD is not sturdy to predict diabetes development among Chinese middle-aged and older populations [5].

Prediabetes according to the IEC guideline was associated with higher risk (hazard ratio: 1.71) of major adverse cardiovascular events compared to normal glucose tolerant (NGT) subjects [6]. Also, prediabetic subjects showed higher levels of total cholesterol (TC) and low-density lipoprotein cholesterol (LDL-c) and were less likely to achieve lipid control with statin therapy and showed poor weight control [7]. Further, cancer risk was higher among PD and T2D population and this risk could not be reduced by dietary and physical activity-based lifestyle interventions [8].

Considering the high risk of diseases associated with the development of T2D, and the controversy between definitions of PD among various races points to the importance of accurate diagnosis of PD especially among high-risk individuals to provide interventions that may prevent the progression to T2D [3].

### 1.1. Objectives

This study tried to evaluate the ability of estimated levels of glycated albumin (GA), HbA1c, C-reactive protein (CRP) and total albumin (TA) to distinguish prediabetic subjects from apparently healthy subjects.

### 1.2. Setting

Department of General Medicine, Faculty of Medicine, Benha University.

### 1.3. Design

Prospective observational non-selective study.

### 1.4. Ethical considerations

The study protocol was introduced for approval by the Faculty Ethical Committee before case collection. The protocol of the study was discussed with the attendants to the blood bank before enrolment, and those accepted to participate signed a written fully informed consent for participation at the end of the case collection. The final approval of the study protocol was obtained in May 2023.

### 1.5. Study population

All subjects who attended the Blood Bank and passed the preliminary clinical and lab evaluation required for blood donation since Aug 2021 were evaluated for exclusion and inclusion criteria. For comparative purposes, the study also included 110 T2D patients of those attending the Diabetes Clinic and 110 cardiac patients of those attending the Cardiology Clinic at The Internal Medicine Department, Benha University Hospital.

### 1.6. Exclusion criteria

Patients younger than 18 and older than 40 years, who had a family history of diabetes mellitus, metabolic syndrome, kidney, liver or cardiac diseases, were excluded from the study. The presence of obesity grade II or III, endocrinopathy, manifestations of protein malnutrition, hypovitaminosis, hemoglobinopathies and maintenance on immunosuppressive drugs are also exclusion criteria.

### 1.7. Inclusion criteria

Apparently healthy subjects who passed the clinical and laboratory evaluation for blood donation and were free of exclusion criteria were included in the study.

### 1.8. Study protocol

All subjects fulfilling the inclusion criteria and enrolled patients were asked to attend the outpatient clinical pathology lab fasting for at least 8-h for giving blood samples and to reattend 1-h and 2-h after receiving a 75-g oral carbohydrate diet to estimate postprandial blood glucose (PPBG). After giving blood samples, all participants were asked to attend the outpatient clinic of Internal Medicine for clinical and ultrasonographic (US) evaluation.

### 1.9. Grouping of the study participants

The volunteers were divided according to the results of the 75-OGTT into NGT if showed FBG <100 mg/dl and normal glucose tolerance on the 75- OGTT, and IGT subjects if showed impaired FBG (IFBG) that was defined according to the Standards of Medical Care in Diabetes as IFBG level in the range of 100–125 mg/dl (Standards, 2010) [9] or IGT as judged by the 75-OGTT.

### 1.10. Blood sampling & investigations

- Fasting blood samples were divided according to the assigned blood variate to be estimated as following:
  1. A sample was collected in fluoride containing tubes till estimation of blood glucose by the glucose oxidase method [10] using BT 1500 Fully Automated Biochemistry Analyzer (Biotechnica Co, Ahmedabad, Gujarat, India).
  2. A sample was added to EDTA for estimation of HbA1c using Latex Turbidimetry (LINEAR CHEMICALS S.L. Joaquim Costa, Montgat, Barcelona, Spain) using IFCC calibrated method depending on the previously that there are small differences observed by quality control and external quality assurance between IFCC-calibrated and NGSP certified methods across a wide HbA(1c) range [11].
  3. Another sample was put in EDTA containing tube and was centrifuged to get plasma for estimation of plasma GA level by a liquid enzymatic method using the Lucica® method for GA, manufactured by Asahi Kasei Pharma Corporation; a specific test for GA (EKF USA, San Antonio, Taxis, USA) according to the manufacturer’s instruction [12].
  4. The remaining part was collected in a plain dry tube, and after clotting was centrifuged 1500 rpm for 20 min to get serum that was divided into two part; one for estimation of serum CRP and TA for calculation of CRP/TA ratio (CAR), and activity levels of aspartate and alanine transaminases (AST and ALT) and lipid profile using BT 1500 Fully Automated Biochemistry Analyzer (Biotechnica Co, Ahmedabad, Gujarat, India) according to the manufacturer’s instructions ^(11)^. The 2nd part of the serum was collected in a clean dry Eppindorff tube and stored at −20 °C until ELISA estimation of serum human insulin using abcam ELISA kits (Cat. No. ab200011, Abcam Inc., San Francisco, USA) [13].
- Two postprandial blood samples were obtained at 1-h and 2-h after taking the 75-g oral glucose diet in fluoride containing tubes for estimation of PPBG-1 and PPBG-2.

### 1.11. Evaluation tools

1. Body mass index (BMI) was calculated as weight (kg) divided by height (m^2^) [14] and was graded according to WHO guidelines as under (UW), average (AW) or over-weight (OW) or obese I–III [15].
2. US detection and grading of steatohepatitis (SH) by the use of the computerized hepatorenal index (HRI) that at cutoff points of 1.49, 1.86, and 2.23 can grade SH as >5–25%, >25-<60%, and ≥60 SH, respectively [16].
3. Diagnosis of non-alcoholic SH (NASH) using the hepatic steatosis index (HSI) that was calculated as: ([ALT/AST ratio] multiplied by Ref. [8]) + BMI + 2 (if the patient was diabetic) + 2 (if the case is female), and the HIS ≥36 is suggestive of NASH [17].
4. Diagnosis of IR using the homeostasis model assessment of IR (HOMA-IR) score that could be calculated according to the suggested equation: ([FBG x FSI]/18)/22.5; HOMAIR >2 suggests the presence of IR [18].
5. The results of the 75-g OGTT were interpreted for diagnosis of glucose intolerance according to the recommendations of the International association of diabetes and pregnancy study groups [19] as follows: FBG ≥92 mg/dl, 1-h BG ≥180 mg/dl and 2-h BG ≥153 mg/dl.
6. Evaluation of the CR according to the AIP that was defined as the base 10 logarithms of the ratio of serum triglyceride (TG) to high-density lipoprotein cholesterol (HDL-c) [20] and was interpreted as low CR if AIP was minus 0.3 to 0.1, medium CR if AIP was >0.1 to 0.24 and high CR if AIP was >0.24 [21].

### 1.12. Study outcomes

1. The primary outcome is the ability of lab investigations to distinguish prediabetic subjects from apparently healthy subjects.
2. The secondary outcomes include
  - The distinguishing the ability of GA, HbA1c levels and the CAR for these prediabetic individuals.
  - The applicability of GA, HbA1c levels and the CAR in conjunction with age, gender and BMI for prediction of IR as judged by HOMA-IR score, hepatosteatosis evaluated by HSI and HRI scores and CR as predicted by the AIP score among the enrolled volunteers.

### 1.13. Statistical analysis

The results were analyzed using SPSS software program (IBM, Ver. 27, 2020, USA) by One-way ANOVA and Chi-square tests. Correlation analysis was applied to evaluate the relation between the presence of PD and its complications as independent variate and constitutional and lab data. The Receiver Operating Characteristic (ROC) curve and Multivariate Regression analyses were applied to define the most significant predictors for PD and its oncoming complications. The area under the curve (AUC) was compared to the area under the reference line to evaluate the significance of the AUC as predictor at 0.05 as a cutoff point for differentiation.

## 2. Results

**Fig. 1:**
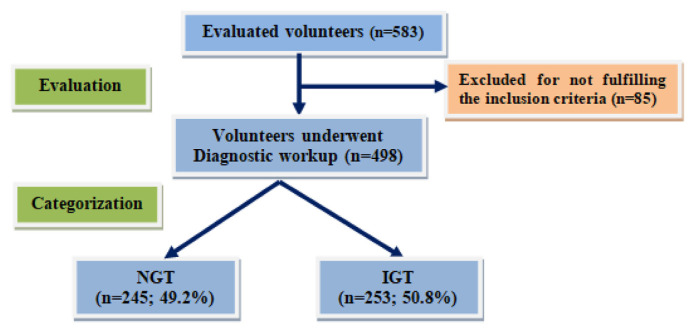
Study flow chart.

Preliminary evaluation excluded 17 subject for being out of age range, another 17 obese of grades II and III, 28 subjects for having liver affection (n = 11), hypertension (n = 6), endocrinopathy (n = 6), kidney affection (n = 5) and 23 individual who did not attend the lab to undergo the required investigations. Among the recruited individuals (n = 498), 245 subjects showed normal glucose tolerance (NGT), while 253 subjects had impaired glucose tolerance (IGT) according to the results of 75-OGTT (Fig. 1).

The estimated FBG, 1-h and 2-h PPBG were significantly higher in samples of T2D patients than all other samples and in samples of subjects of IGT than in NGT groups. The estimated levels of FBG and 2-h PPBG were significantly higher in samples of cardiac patients than in samples of volunteers, while the levels estimated at 1-h were significantly higher than that of NGT subjects. Subjects of the NGT group were found to be significantly younger, had significantly lower BMI and were mainly males than IGT subjects and T2D patients. IGT subjects and T2D patients were mainly females, older and had higher BMI than other participants as shown in details in Table 1.

**Fig. 2:**
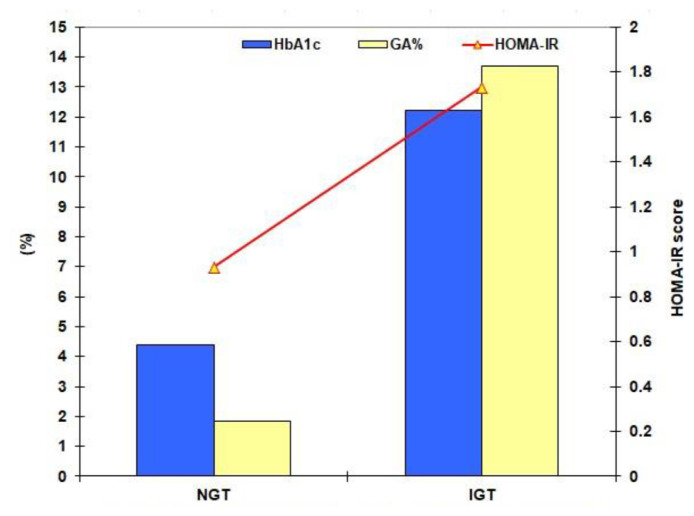
Mean levels of HbA1c and GA, and HOMA-IR score of the screened volunteers categorized according to the results pf 75-OGTT.

The mean value of the estimated HbA1c levels was non-significantly higher, while the mean values of the estimated GA% and HOMA-IR score were significantly higher in IGT than in NGT subjects (Fig. 2). Compared to T2D patients, the estimated glycemic indices were significantly lower in volunteers and cardiac patients. Further, 69 (27.3%) IGT subjects, 73 (66.4%) T2D patients and 31 (28.2%) cardiac patients were insulin resistant, while no NGT subject was insulin resistant (Table 2).

Mean levels of serum TC, LDL-c and TG were significantly lower and serum HDL-c levels were significantly higher in samples of NGT than in IGT subjects and in patients’ samples than in volunteers’ samples with significant differences between patients’ samples. The estimated serum CRP levels were significantly lower in samples of NGT subjects compared to levels estimated in all other samples and were significantly higher in samples of diabetics than in samples of IGT subjects and cardiac patients with significantly higher levels in samples of cardiac patients. Total albumin levels were significantly higher in samples of NTG subjects than in other samples and samples of IGT subjects than in patients’ samples. Serum levels of AST were significantly higher, while serum ALT levels were significantly lower with a significantly higher AST/ALT ratio in NGT samples than other samples. Regarding samples of IGT subjects and patients, AST/ALT ratios were significantly lower in cardiac patients than in IGT subjects and diabetics with non-significant differences between the latter participants (Table 2).

**Fig. 3:**
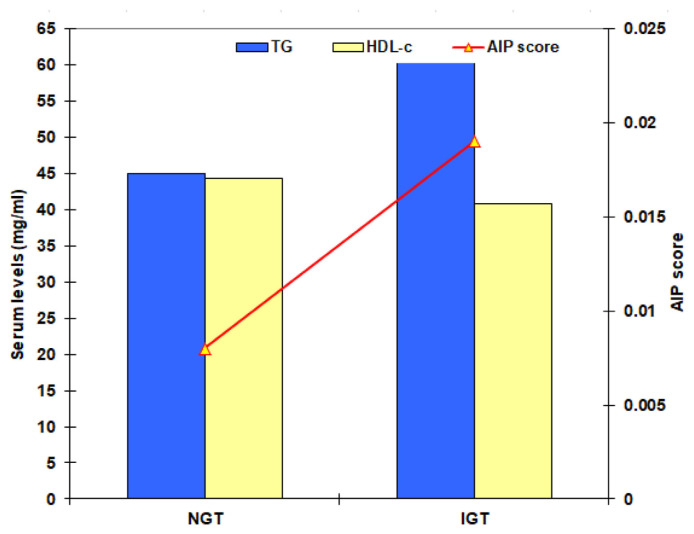
Mean serum levels of TG and HDL-c and AIP score of the screened volunteers categorized according to the results of 75-OGTT.

**Fig. 4:**
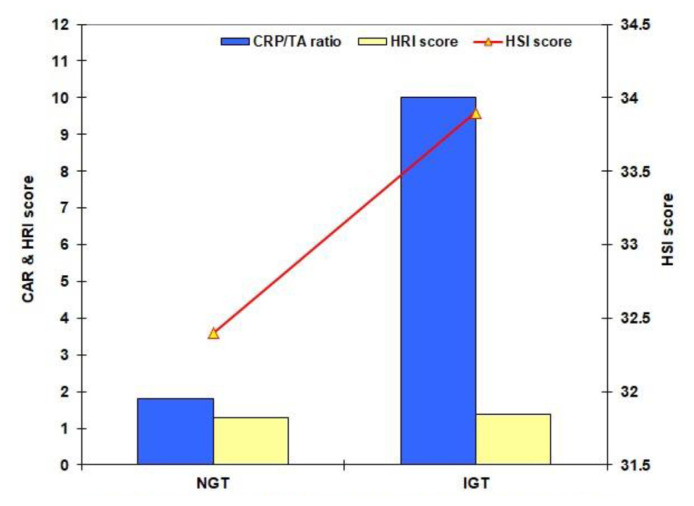
Mean levels of CRP/TA ratio (CAR), HRI and HSI scores of the screened volunteers categorized according to the results of 75-OGTT.

The calculated AIP score was significantly lower in volunteers’ than in patients’ groups and NGT than IGT groups with a significantly higher frequency of patients had AIP<0.1 among volunteers than patients and among NGT than IGT subjects (Table 3, Fig. 3). The calculated CAR was significantly lower in NGT than other participants and in IGT than in diabetics and cardiac patients who showed nonsignificant difference. The calculated HSI and HRI scores and the frequency of patients with steatohepatitis were significantly lower in NGT subjects and were significantly higher in diabetics than IGT subjects and cardiac patients who showed non-significant differences (Table 3, Fig. 4).

**Fig. 5:**
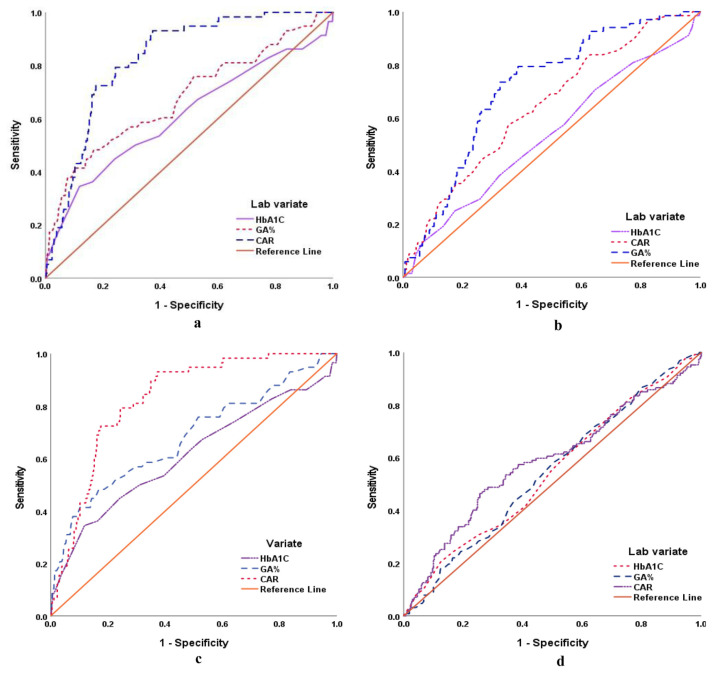
a. ROC Curve for Lab predictors for PD as presence of impaired glucose tolerance. b. ROC Curve for Lab predictors for HOMAjudged by IR score>2. c. ROC Curve for Lab predictors for AIP. d. ROC Curve for Lab predictors for HSI score>0.1 score>36.

The calculated AIP (r = 0.815, p < 0.001), HOMA-IR (r = 0.786, p < 0.001), HSI (r = 0.205, p < 0.001) and HRI (r = 0.229, p < 0.001) scores showed positive significant correlation with the presence of PD as manifested by IGT. ROC curve analysis defined older age and high BMI as the most significant predictors for the presence of PD state and high AIP score, while high BMI and female gender as the most significant predictors for HSI score >36, while high BMI as the most significant predictor for HOMA-IR>2 and high HRI score. Regarding the lab variate high GA% and CAR could predict PD (Fig. 5a), HOMA-IR>36 (Fig. 5b) and AIP score >0.1 (Fig. 5c), while high CAR is the only predictor for HS as judged by HSI score >36 (Table 4, Fig. 5d).

## 3. Discussion

Depending on the results of the 75-OGTT applied for 489 volunteers, the prevalence of impaired glucose tolerance (IGT) was 50.8% and was in ranges recently documented; 3.92% and 47.06% according to the user definition ^(6)^ or 67% according to ADA criteria ^(4)^. Furthermore, the current study detected a positive significant correlation between the presence of PD and high HOMA-IR, HSI, HIR and AIP scores. These findings indicated that PD is a risk for further dysglycemia with its subsequent complications, especially the hepatic and cardiac complications, and assured the necessity for early distinguishing prediabetic subjects among apparently healthy individuals to prevent the progression of prediabetes to T2DM.

Similarly, **Kirthi et al**. [22] detected progressive full-thickness macular thinning with increasing dysglycemia across the normal glucose tolerant (NGT), IGT and T2D groups and concluded that progressive dysglycemia is associated with macular thinning before the onset of visible retinopathy. Also, **Choi et al**. [23] found prediabetics are more prone to develop IR and possible progression to diabetic nephropathy. Thereafter, **Blond et al**. [3] reported that PD is a highly prevalent risk factor for T2D and is associated with diabetic complications and mortality and **Cui et al**. [4] found PD was significantly associated with a higher risk of major adverse cardiovascular event than normoglycemia. These studies insisted on the necessity for early detection of IGT among people and recently the Behavioral Nudges for Diabetes Prevention trial provided evidence about the effectiveness of interventions focused on diabetes prevention through diagnosis of prediabetics [24].

The estimated GA% showed significant variation across the NGT, IGT and T2D groups, a positive significant correlation with the presence of IGT and statistical analyses defined high GA% as a significant predictor for dysglycemia and IGT among apparently healthy individuals. Similarly, **Bai et al**. [25] found the unadjusted hazard ratios for both diabetes and PD increased proportionally with increasing GA levels in a dose–response manner and AUC for GA, FBG and 2-h PPBG for prediction of DM showed non-significant differences, so concluded that GA is of good prognostic utility in predicting diabetes.

In support of the diagnostic value of GA% for dysglycemia, **Kengne et al**. [26] detected improved diagnostic ability for dysglycemia when coupling the estimation of GA to the estimation of HbA1c than depending on HbA1c alone. Also, **Shao et al**. [27] found the sensitivity and specificity for a combination of OGTT, HbA1c and GA is 100% and 90.11% for NGT; 75.11% and 97.32% for IGT; and 97.14% and 100%, and 94.67% and 100% for hypoglycemia and DM.

High GA% showed a significant association with AIP score and HOMA-IR and could predict cardiac risk and IR in the studied cohort of PD, and these findings indicated the predictive ability of GA% for complications among prediabetics. Following these findings, L**ee et al**. [28] found the development of early neurological deterioration and poor functional status in PD increased progressively with increased GA levels and concluded that pre-stroke dysglycemia estimated by GA was associated with these events after ischemic stroke in prediabetic patients. During the COVID-19 outbreak, the increased severity of disease in apparently healthy people was attributed to the preference of SARS-CoV-2 for GA that was detected in these patients who developed complications [29]. Further, **Noguchi-Shinohara et al**. [30] found higher GA levels in prediabetics were associated with the development of Alzheimer’s disease.

The estimated serum CRP levels were significantly higher and total albumin levels were significantly lower with subsequently higher CRP/TA ratio (CAR) in diabetic and cardiac patients than in the volunteer group and IGT than NGT volunteers. Moreover, CAR was positively correlated with uncontrolled hyperglycemic status as judged by high HbA1c%. Furthermore, statistical analyses showed the ability of the high CAR to differentiate between IGT and NGT subjects and to predict IR, steatohepatitis and cardiac risk among IGT subjects.

These data go in hand with **Bulut & Avci** [31], who detected higher carotid intima thickness with impaired glucose metabolism in prediabetic and newly-diagnosed T2D patients than normoglycemic subjects and found high CRP, LDL-c and low HDL-c levels were independent risk factors for increased intimal thickness. Also, **Demirkol et al**. [32] thought that CAR, which reflects the liver-related inflammatory status, could be applied as an inflammation marker in both PD and DM to evaluate the deregulated local hepatic inflammatory status. Further, **Gedebjerg et al**. [33] during follow-up of patients with recent T2DM reported a positive relation between high CRP levels and the risk of cardiovascular events and found high CRP level is a stronger prognostic biomarker of all-cause mortality and might be used to improve early detection and prevention of deadly diseases other than cardiovascular events.

In support of the applicability of CAR for the prediction of cardiac risk, recent studies reported a significant relationship between high CRP levels and all-cause death in patients with STEMI [34], new-onset cardiac events or stroke [35] and **Liao et al**. [36] found both of AIP score and CAR are independent predictors for the occurrence of coronary microvascular disease. Following the relation between high CAR and diabetes complications, **Sant**’**Ana et al**. [37] found the mortality risk during 6-m of hemodialysis proportionate with the CAR among these patients and **Zengin et al**. [38] detected a higher risk for major amputations for diabetic foot patients with high CAR.

Furthermore, statistical analyses defined high BMI, female gender and older age as significant predictors for PD, and are risk factors for the development or progression of IR, steatohepatitis and increased cardiac risk as judged by high AIP that positively correlated with the presence of PD. In line with these findings, **Silver et al**. [39] detected an association between obesity and PD, and found caloric restriction alone for these individuals resulted in decreased body weight with improved HOMA-IR score. Also, **Shaheen et al**. [40] found the prevalence of NASH was increased across populations of NGT, PD and DM and was 30.5%, 56.4% and 82.6%, respectively. Moreover, **de Ritter et al**. [41] found BMI, fat/lean mass and hip circumference increased with PD and DM than normoglycemia in females than in males.

## 4. Conclusion

Prediabetes is not uncommon and may pass unnoticed to end at T2D. Both high GA% and CAR might differentiate prediabetics among the apparently healthy population. These biomarkers could predict increased risk for IR, steatohepatitis and cardiac risk among prediabetics, especially obese and older subjects. Females are more vulnerable to prediabetes and more liable to develop complications of this state.

## Figures and Tables

**Table 1:** The results of the 75-OGTT and demographic data of the study participants.

Variate	Group	NGT (n = 245)	IGT (n = 253)	T2D (n = 110)	Cardiac (n = 110)
FBG (mg/dl)		78.2 ± 6.4	103.5 ± 18.2^a^	145.5 ± 13^a^,^b^	83.1 ± 8.5^a^,^b^,^c^
1-h PPBG (mg/dl)		150.4 ± 12.1	163.3 ± 9.5^a^	245.4 ± 33.4^a^,^b^	166.6 ± 14.6
2-h PPBG (mg/dl)		125.6 ± 6.9	132.8 ± 9.9^a^	208 ± 40.3^a^,^b^	139.7 ± 16.2^a^,^b^,^c^
Age (years)	<25	99 (40.4%)	57 (22.5%)^a^	7 (6.4%)^a^,^b^	0^a^,^b^,^c^
	25–29	88 (35.9%)	56 (22.1%)	70 (63.6%)	33 (30%)
	30–34	39 (15.9%)	86 (34%)	30 (27.3%)	65 (59.1%)
	≥35	19 (7.8%)	54 (21.3%)	3 (2.7%)	12 (10.9%)
	Mean (±SD)	26.3 ± 5.2	30.1 ± 5.4^a^	29 ± 2.7^a^	31.2 ± 2.8^c^
Gender	Males	177 (72.2%)	99 (39%)^a^	31 (28.2%)^a^,^b^	73 (66.4%)^b^,^c^
	Females	68 (27.8%)	154 (61%)	79 (71.8%)	37 (33.6%)
BMI (kg/m^2^)	Underweight	9 (3.7%)	17 (7.1%)^a^	0^a^,^b^	0^a^,^b^,^c^
	Average	33 (13.5%)	30 (22.3%)	0	0
	Overweight	175 (71.4%)	96 (61.6%)	20 (16.8%)	96 (22.4%)
	Obese	28 (11.4%)	110 (8.9%)	90 (75.9%)	14 (63.8%)
	Mean (±SD)	27.8 ± 2.8	29.2 ± 4.1^a^	31.6 ± 1.9^a^,^b^	29 ± 1.8^a^,^c^

a Indicates the significance versus NGT.

b Indicates the significance between T2D and cardiac patients in comparison to IGT subjects.

c Indicates the significance between T2D and cardiac patients.

**Table 2:** Lab data of the study participants.

Lab variate	Group		NGT (n = 245)	IGT (n = 253)	T2D (n = 110)	Cardiac (n = 110)
Glycemic status	HbA1c (%)		4.38 ± 0.37	4.53 ± 0.55	5.24 ± 0.66^a^,^b^	4.65 ± 0.54^a^,^c^
	GA (%)		12.2 ± 1.32	13.7 ± 2^a^	15.13 ± 2.28^a^,^b^	12.5 ± 1.9^b^,^c^
	HOMA-IR score	Score<2	245 (100%)	184 (72.7%)^a^	37 (33.6%)^a^,^b^	79 (71.8%)^a^,^c^
		score≥2	0	69 (27.3%)	73 (66.4%)	31 (28.2%)
		Mean	0.93 ± 0.22	1.73 ± 0.38^a^	2.12 ± 0.43^a^,^b^	1.54 ± 0.47^a^,^b^,^c^
Lipid profile	TC (mg/ml)		173.6 ± 14.9	180.9 ± 16.1^a^	210 ± 26.5^a^,^b^	202 ± 23.6^a^,^b^,^c^
	LDL (mg/ml)		120.4 ± 15.6	127.9 ± 17.6^a^	158.6 ± 28^a^,^b^	150.1 ± 23.5^a^,^b^,^c^
	TG (mg/ml)		45 ± 4.9	61.5 ± 7.7^a^	65.8 ± 9.6^a^,^b^	64.5 ± 6.1^a^,^b^
	HDL-c (mg/ml)		44.2 ± 4.1	40.8 ± 5.6^a^	38.2 ± 5.1^a^,^b^	39 ± 5.3^a^,^b^
CRP (IU/ml)			24.4 ± 15.8	28.9 ± 16.7^a^	81.3 ± 16.2^a^,^b^	77.3 ± 12.9^a^,^b^,^c^
Total albumin (g/ml)			4.72 ± 0.4	4.42 ± 0.56^a^	4.2 ± 0.2^a^,^b^	4.15 ± 0.6^a^,^b^
Liver enzymes	AST (IU/ml)		16.6 ± 2.9	15.3 ± 2.9^a^	14.9 ± 3.1^a^	15.7 ± 3.7
	ALT (IU/ml)		33.6 ± 4.1	40 ± 7.2^a^	36 ± 7.9^a^	43 ± 7.6^a^,^b^,^c^
	AST/ALT ratio		4 ± 0.8	3.43 ± 0.83^a^	3.47 ± 0.82^a^	3 ± 0.79^a^,^b^,^c^

a Indicates the significance versus NGT.

b Indicates the significance between T2D and cardiac patients in comparison to IGT subjects.

c Indicates the significance between T2D and cardiac patients.

**Table 3:** The calculated scores of the study participants.

Lab variate Group	NGT (n = 245)	IGT (n = 253)	T2D (n = 110)	Cardiac (n = 110)
AIP score	0.008 ± 0.05	0.19 ± 0.08^a^	0.23 ± 0.11^a^,^b^	0.22 ± 0.07^a^,^b^
Frequency of AIP<0.1	234 (95.5%)	227 (89.7%)^a^	15 (13.6%)^a^,^b^	4 (3.6%)^a^,^b^,^c^
CRP/TA ratio (CAR)	1.8 ± 0.59	10 ± 5.8^a^	19.5 ± 4.9^a^,^b^	19 ± 4.2^a^,^b^
HSI score	32.4 ± 3.2	33.9 ± 4.1^a^	38.5 ± 2.2^a^,^b^	33.9 ± 2.3^a^,^c^
Frequency of HSI<36	204 (83.3%)	168 (66.4%)^a^	21 (19.1%)^a^,^b^	86 (78.2%)^b^,^c^
HRI score	1.29 ± 0.16	1.38 ± 0.23^a^	1.56 ± 0.21^a^,^b^	1.35 ± 0.19^a^,^c^

a Indicates the significance of difference versus NGT.

b Indicates the significance of the difference between T2DM and cardiac patients in comparison to IGT subjects.

c Indicates the significance of the difference between T2DM and cardiac patients.

**Table 4:** The ROC curve analysis for the predictors of the presence of PD and possible complications.

Variate		AUC	SE	P	95% CI	Variate	AUC	SE	P	95% CI
**PD**	**Age**	0.744	0.033	<0.001	0.679–0.809	**HbA1c**	0.611	0.045	0.006	0.523–0.699
	**Gender**	0.589	0.040	0.027	0.512–0.667	**GA**	0.827	0.024	<0.001	0.780–0.874
	**BMI**	0.347	0.037	<0.001	0.274–0.420	**CAR**	0.679	0.042	<0.001	0.597–0.761
**HOMA-IR** >**2**	**Age**	0.400	0.033	0.008	0.335–0.464	**HbA1c**	0.534	0.039	0.364	0.457–0.611
	**Gender**	0.616	0.036	0.002	0.545–0.687	**GA**	0.715	0.030	<0.001	0.655–0.774
	**BMI**	0.668	0.037	<0.001	0.596–740	**CAR**	0.643	0.035	<0.001	0.575–0.712
**HSI score** >**36**	**Age**	0.541	0.030	0.164	0.483–600	**HbA1c**	0.544	0.030	0.135	0.486–0.603
	**Gender**	0.613	0.028	<0.001	0.558–0.669	**GA**	0.544	0.029	0.139	0.487–0.601
	**BMI**	0.639	0.028	<0.001	0.584–0.696	**CAR**	0.585	0.031	0.004	0.524–0.646
**HRI**	**Age**	0.458	0.031	0.204	0.398–0.519	**HbA1c**	0.556	0.034	0.087	0.490–0.623
	**Gender**	0.530	0.033	0.359	0.466–0.595	**GA**	0.552	0.033	0.115	0.487–0.617
	**BMI**	0.651	0.030	<0.001	0.593–0.709	**CAR**	0.626	0.032	<0.001	0.563–0.689
**AIP score** > **0.1**	**Age**	0.744	0.033	<0.001	0.679–0.809	**HbA1c**	0.611	0.045	0.006	0.523–0.699
	**Gender**	0.589	0.040	0.027	0.512–0.667	**GA**	0.679	0.042	<0.001	0.597–0.761
	**BMI**	0.347	0.037	<0.001	0.274–0.420	**CAR**	0.827	0.024	<0.001	0.780–0.874

Multivariate regression analysis of the studied variate defined older age and high GA% as the persistently significant predictors (β = 0.276 & 0.378, respectively; P < 0.001) for the presence of PD. Also, high BMI and GA% were found to be the most significant (β = 0.210 & 0.228, respectively; P < 0.001) predictors for HOMA-IR score>2, while high BMI and CAR for prediction of steatohepatitis scores; HSI (β = 0.245, P < 0.001 & 0.142, P = 0.001) and HRI (β = 0.202 & 0.168, respectively; P < 0.001). For prediction of AIP score>0.1, old age (β = 0.282, P < 0.001), high BMI (β = 0.168, P < 0.001), high GA% (β = 0.192, P < 0.001) and high CAR (β = 0.340, P < 0.001) are the most significant predictors.

## Data Availability

Data is available when requited.
